# Improving Data Quality with an Accumulated Reputation Model in Participatory Sensing Systems

**DOI:** 10.3390/s140305573

**Published:** 2014-03-20

**Authors:** Ruiyun Yu, Rui Liu, Xingwei Wang, Jiannong Cao

**Affiliations:** 1 Software College, Northeastern University, No. 11, Lane 3, Wenhua Road, Heping District, Shenyang 100819, China; 2 Department of Computing, Hong Kong Polytechnic University, Hung Hom, Kowloon, Hong Kong, China; E-Mails: csrliu@comp.polyu.edu.hk (R.L.); csjcao@comp.polyu.edu.hk (J.C.); 3 College of Information Science and Engineering, Northeastern University, No. 11, Lane 3, Wenhua Road, Heping District, Shenyang 100819, China; E-Mail: wangxw@mail.neu.edu.cn

**Keywords:** participatory sensing, reputation, contribution, data quality

## Abstract

The ubiquity of mobile devices brings forth a sensing paradigm, participatory sensing, to collect and interpret sensory information from the environment. Participants join in multifarious sensing tasks and share their data. The sensing result can be obtained in light of shared data. It is not uncommon that some corrupted data is provided by participants, which makes sensing result unreliable accordingly. To address this nontrivial issue, we proposed the accumulated reputation model (ARM) to improve the accuracy of the sensing result. In ARM, participants' reputation will be computed and accumulated based on their sensing data. The sensing data from reputable participants make higher contributions to the sensing result. ARM performs well on calculating accurate sensing results, even in extreme scenarios, where there are many inexperienced or malicious participants.

## 1. Introduction

Taking advantage of increasing storage resources, powerful computing capacity, high-quality networks and sophisticated embedded sensors, ever-more capable mobile devices promise to provide a myriad of services, such as data collection and integration, information sharing and social networking. Thus, a novel sensing paradigm, participatory sensing, appeared on the scene [1]. A general doctrine of participatory sensing is that individuals and communities use mobile devices to collect and analyze data for use in discovery [2].

The inherent mobility of participants provides unprecedented spatiotemporal coverage and also makes it possible to observe unpredictable events. Moreover, by including people in the sensing loop, it is now possible to design applications that can dramatically improve the daily lives of individuals and communities.

In general, there are two main groups of participatory sensing applications, environment-centric applications (air pollution [3], noise [4], traffic [5] and scenery [6]) and user-centric applications (social network [7] and user activity [8]). The former ones mainly monitor, record and interpret environmental information; the latter ones rely on the user data from mobile devices, and valuable information is produced through analysis.

Several universities and institutes have done relevant research in this area and several exciting participatory sensing applications have emerged in recent years. PEIR [9] is an application that uses location data sampled from everyday mobile phones to calculate personalized estimation of environmental impact and exposure. CarTel [10] is a mobile sensor computing system designed to collect, process, deliver and visualize data from sensors located on mobile units, such as automobiles. Lu *et al.* [11] proposed bubble-sensing, a new sensor network abstraction that allows mobile phone users to create a binding between tasks (e.g., take a photo or sample audio every hour indefinitely) and the physical world at locations of interest, which remains active for a duration set by users.

As we mentioned before, research in participatory sensing still remains at the theoretical and experimental level, which focuses on how to design attractive and beneficial applications. A few works devote themselves to enhance the quality of sensing data captured by participants in participatory sensing applications. In reality, participatory sensing cannot occur as expected if the sensing data is unreliable or inaccurate.

In a participatory sensing system, the sensing result highly relies on the sensing data collected by mobile devices carried by participants. However, it is arduous for the system to obtain accurate sensing data, because high mobility and environmental complexity in participatory sensing systems may bring much more uncertainty, and there may be some inexperienced and malicious participants who will generate corrupted sensor data. For example, the location and position of devices have great effects on the final sensing data. It is routine for people to put their mobile devices in a pocket or bag, but in this case, the devices will provide inaccurate data when they are used to monitor air pollution. Furthermore, malicious participants falsify sensing data and degrade the quality of the sensing result. Therefore, it is indispensable to identify corrupted data and improve the accuracy of the sensing result.

To address this non-trivial issue, we propose the accumulated reputation model (ARM) for improving sensing quality in environmental participatory sensing systems. ARM first analyzes sensing data provided by participants and then evaluates the trustworthiness of participants using an accumulated reputation score, which can minimize the effects of corrupted data and eventually achieve a high accuracy result.

Our contributions are presented as follows:

- The proposed reputation mechanism to evaluate the trustworthiness of participants is as follows. Each participant's reputation score is calculated based on the quality of sensing data and the frequency of participation. Additionally, the contribution score is proposed to estimate the quality of the sensing data provided by each participant in the current sensing activity, and the reputation score can present the accumulation of historical participation.
- If there is no sufficient number of participants in a sensing application, normal participants (the participants who collect accurate data) probably account for a small proportion of the total. This will lead to imprecise results, because of the overwhelming influence of corrupted data generated by those abnormal participants. ARM will alleviate such bad effects and improve the quality of the sensing result.

The reminder of this paper is organized as follows. Relevant research works are presented in Section 2. Subsequently, the proposed mechanism, ARM, is elaborated in Section 3. In Section 4, ARM is evaluated in various scenarios. We conclude with a summary of our contributions in Section 5.

## 2. Related Work

The reputation system has a long history and is by no means a fad of only one research area. It has been widely used for comment rating environments [12], such as Taobao and Amazon. Taobao established their own reputation system to enhance buying and selling experiences [13]. For example, buyers assign one to five stars to rate the commodities and sellers based on their satisfaction. This approach is easy to implement and understand, but with some drawbacks. Firstly, negative ratings can be easily drowned out by a large pool of positive ratings. Secondly, it is easy for system administrators to change ratings illegally. This approach is not viable in the context of participatory sensing systems.

Drawing the inspiration from the comment rating environment, the reputation system is also applied in ad hoc wireless networks [14,15]. Michiardi and Molva [14] proposed a generic mechanism based on reputation to enforce the cooperation among the nodes of mobile *ad hoc* networks to prevent selfish behavior. In [15], Bayesian analysis is used to formulate a similar problem, and the resulting reputation systems are shown to counter any misbehaving nodes. Bayesian reputation systems can be adapted with relative ease in different types of applications and environments [16]. For example, the reputation framework, RFSN [17] makes use of beta reputation [16] for associating a reputation score with each sensor node in a traditional embedded wireless sensor network. Beta reputation has simple updating rules, as well as it facilitates the easy integration of aging. However, it takes a less aggressive approach in penalizing participants that contribute corrupted data. It should be noted that, in participatory sensing applications, the period over which a participant may contribute corrupted data may potentially be short-lived.

From a perspective of security, the reputation system has been widely advocated as an effective mechanism for distributed and intelligent environments. Moya *et al.* [18] proposed a reputation system in the wireless sensor network, which allows bad reputation feedback to effectively detect and confine some common attacks. Rather than focusing on deploying a reputation system in a wireless sensor network, Moya *et al.* [19] proposed a reputation mechanism in supervisory control and data acquisition (SCADA) sensor networks, which can achieve fault tolerance and enhanced resistance to some unknown attacks. The proposed mechanism enhanced with distributed agents using an unsupervised type of neural network (*i.e.*, Kohonen networks). In a more general intelligent environment, a bio-inspired enhancement of the reputation system is applied to achieve better performance of security [20]. However, unlike in a sensor network or a P2P network, there is no explicit node and fixed topological structure in a participatory sensing system. Actually, the information from the environment is collected and shared through mobile devices carried by participants rather than deployed sensors. Therefore, the advantages against attacks in a wireless sensor network, including redundancy, continuous adaptation and relation between nodes, have passed out of existence. Hence, an appropriate reputation mechanism needs to be proposed to cater to participatory sensing.

To our knowledge, little attention has gone to reputation in participatory sensing systems. Huang *et al.* [21] implements a system in noise monitoring to identify corrupted noise data. However, the system focuses on the data provided by participants in the current monitoring application without considering the accumulated reputation of participants, and the system is based on a situation for which normal data always accounts for the majority. Hence, the system cannot produce accurate results if corrupted data make up the biggest part of the total data. Yang *et al.* [22] established a reputation management system in participatory sensing for data classification and provided information for campaign organizers and data analysts to facilitate their decisions. However, the accuracy of the sensing result is not their interest.

## 3. Accumulated Reputation Model

In this section, we propose and elaborate the accumulated reputation model (ARM) in the context of a participatory sensing system.

### 3.1. Overview

**Figure 1.**
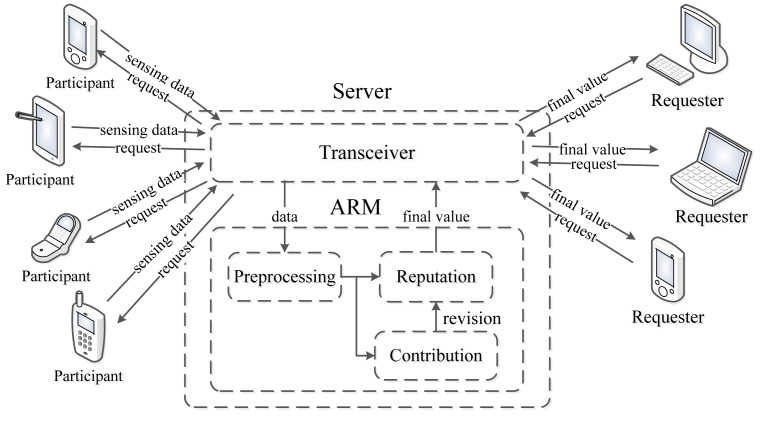
Participatory sensing using the accumulated reputation model (ARM).

Figure 1. depicts the framework of a participatory sensing application using ARM.

Generally, requesters (the request sent by a PC, laptop or smart mobile device) send a sensing request to the participants through a server in a participatory sensing application. After sensing, each participant uploads sensing data to the server through a transceiver module, and the ARM residing in the server processes all the data obtained from the participants to produce a sensing result. Finally, the server sends back the final result to the requesters.

More specifically, the ARM consists of three phases: preprocessing, computing the contribution score and computing the reputation score. In the preprocessing part, the density-based outlier detection algorithm [23] is adopted to identify corrupted sensing data, which is deemed too distant from the majority of the data. Afterwards, the ARM generates the contribution score of each participant in light of their sensing data. Subsequently, the reputation score is calculated based on the historical contribution score. Meanwhile, every participant updates its reputation score using a per-round contribution score. Finally, the ARM generates a sensing result for the requesters in a participatory sensing application.

### 3.2. Model Design

The participants provide not only sensing data, but also additional information, such as spatial and temporal information, current time, *etc.* In this work, we assume that the uploaded data is represented as a five tuple < *id, sensing data, temporal data, spatial data, additional data* >. The *id* is the unique identifier of each device. The *sensing data* is the data captured from the environment by participant *i*. *Temporal data*, normally, is the time point when data is sensed, and *spatial data* represents the location information, where the data is captured. Furthermore, *additional data* consists of the information required by a particular participatory sensing application. To get more accurate sensing data, each participant monitors environmental phenomena for successive equal time slots. For better understanding, the main notations are presented in Table 1.

In an ideal environment, participants provide accurate sensing data, and the sensing result is obtained by analyzing all the data. Unfortunately, there may be inexperienced and malicious participants, which provide corrupted sensing data in the participatory sensing system. Therefore, we design a *preprocessing* part to identify corrupted data from abnormal participants (the ones who generate corrupted or malicious data).

#### 3.2.1. Preprocessing

Usually, the number of normal participants is larger than that of abnormal participants in a large sensing field, so we choose the density-based outlier detection algorithm proposed in [24] to preprocess the sensing data, *s_i_*, accepted from each participant. The details are illustrated in Equations (1) and (2).

(1) $$A=\overset{n}{\underset{i=1}{\text{∑}}}m_{i}\times s_{i}$$

(2) $$m_{i}=\frac{\frac{1}{\frac{{\left(s_{i}-A\right)}^{2}}{{\text{∑}}_{i=1}^{n}{\left(s_{i}-A\right)}^{2}+\in }}}{{\text{∑}}_{j=1}^{n}\frac{1}{\frac{{\left(s_{i}-A\right)}^{2}}{{\text{∑}}_{i=1}^{n}{\left(s_{i}-A\right)}^{2}+\in }}}$$

As shown in Algorithm 1, the algorithm, in nature, is iterative. At first, it is initialized $m_{i}=\frac{1}{n}$. *A* and *m_i_* are computed in each iteration. $m_{i}^{f}$ equals to $m_{i}^{t}$ when the convergence $\left|m_{i}^{t}-m_{i}^{t-1}\right|<\eta$ is observed in the *t*-th iteration.

**Algorithm 1**: Preprocessing in ARM.
**Input:** Number of participant *N* = {1, 2,…, *n*}, sensing data *S* = {*s*_1_, *s*_2_, …, *s_n_*} of *n* participants**Output:**
$M=\{m_{1}^{f},m_{2}^{f},\ldots ,m_{n}^{f}\}$**1****for**
*i* = 1 *to n*
**do****2** *M_i_* ← *initial_value*;**3** **while**
*convergence*
**do****4**  Compute *A* using Equation (1);**5**  **for**
*t* = 1 *to l*
**do****6**   Compute 
$m_{i}^{t}$ using Equation (2);**7**  **end****8**  convergence 
$\leftarrow m_{i}^{t}-m_{i}^{t-1}(m_{i}^{0}=m_{i})$;**9** **end****10** 
$m_{i}^{f}\leftarrow m_{i}^{t}$**11****end**
It is obvious that stricter convergences could be chosen to produce more accurate results according to specific scenarios. *∊* is a small positive constant which is needed to improve the algorithm's numerical properties, and more discussions are shown in [24].

#### 3.2.2. Contribution Score

After *preprocessing*, ARM detects corrupted data that deviate from the majority of sensing data, and *m_i_* is calculated as a weight according to the sensing data provided by each participant.

**Figure 2.**
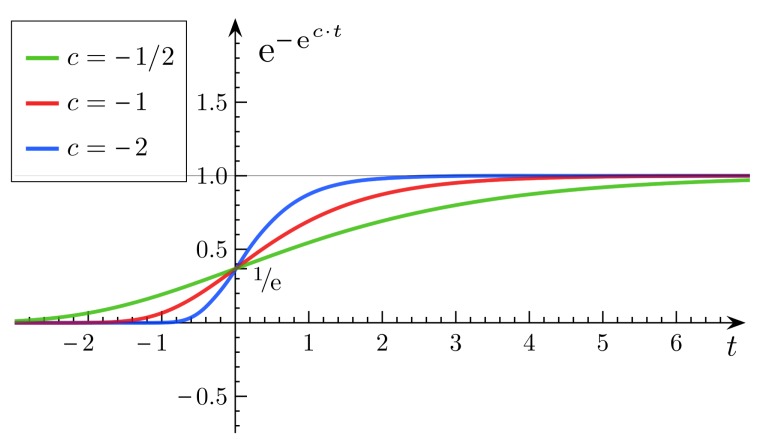
Gompertz function $ae^{be^{cM_{i}^{f}}}$ (a = 1, b = −1).

In order to obtain a contribution score of each participant, the Gompertz function [25] is adopted to produce the contribution score *C* = {*c*_1_, *c*_2_, …, *c_n_*} for participants. A Gompertz function (also called a Gompertz curve), named after Benjamin Gompertz, is a sigmoid function, which originates from population growth (as shown in Figure 2). It is a type of mathematical model for a time series, where growth is slowest at the start and the end of a time period. The right-hand or future value asymptote of the function is approached much more gradually by the curve than the left-hand or lower value asymptote, in contrast to the logistic function in which both asymptotes are approached by the curve symmetrically.

The Gompertz function has the following features: (1) the curve will be approaching an asymptote, but it will never go beyond the extreme. (2) the variation of the curve is gradual, smooth, but not abrupt. (3) the maximum value of the curve is approaching the extreme, and the growth rate falls exponentially with the current size until zero.

Therefore, *m_i_* can be obtained through the outlier detection algorithm. The lower the *m_i_*, the higher the degree of isolation, and *vice versa*. At first, we assume the extreme of the Gompertz function, which is the maximum reputation score of one. The Gompertz function has three phases, which are the reputation doubting phase (beginning), the rapid growth of the reputation phase (middle) and, lastly, the good reputation phase (end). The *m_i_* will be mapped to the x-axis through the normalization method. If *m_i_* is low, this means that the participant is in the reputation doubting phase and has a low reputation. If *m_i_* is in the middle range, this means that the participant is recognized as a normal participant, and its reputation will grow rapidly with the increase of *m_i_*, so that participant *i* can gain a better reputation quickly. Lastly, if *m_i_* is high enough, this means that the participant is prestigious in this participatory sensing application. This can be represented by the last phase of the Gompertz function, where the corresponding reputation value of *m_i_* is approaching an ideal value.

*c_i_* can be computed by the Gompertz function as in Equation (3).

(3) $$c_{i}=a\times e^{b\times e^{c\times m_{i}^{\text{norm}}}}$$

where *a* is the upper asymptote, coefficients *b* and *c* are negative numbers (*b* sets the x displacement; *c* sets the growth rate (x scaling)) and *e* is Euler's number (*e* = 2.71828…). As shown in Equation (4), this is nomalized in order to fall into the interval [−1, 1].

(4) $$m_{i}^{\text{norm}}=\frac{2(m_{i}-\min{\left\{m_{i}\right\}}_{t=1}^{n})}{\max{\left\{m_{i}\right\}}_{t=1}^{n}-\min{\left\{m_{i}\right\}}_{t=1}^{n}}$$

where $\max{\left\{m_{i}\right\}}_{t=1}^{n}$ and $\min{\left\{m_{i}\right\}}_{t=1}^{n}$ represent the maximum and minimum mutual credit in participation, respectively.

#### 3.2.3. Reputation Score

In the *preprocessing* and *computing contribution score* parts, the majority of participants who provide similar sensing data will get a higher contribution score, namely they will make more contributions to the result. It takes it common knowledge that most participants generate relatively accurate sensing data.

However, in particular circumstances, the number of abnormal participants would be larger than that of normal ones in the sensing field, which will decrease the effects of accurate data and calculate final a value based on corrupted data. This may lead to a fatal disaster when making decisions based on such corrupted data in a participatory sensing system.

Hence, we introduce *reputation score* to overcome this drawback and improve the sensing result quality. The contribution score of participant *i* is generated in each act of participation. After *k* times of participation, a participant will show its reputation value based on its historical behaviors. It will be more effective if such a reputation score is introduced to calculate the participants' contributions.

The reputation score *R* = {*r*_1_, *r*_2_, …, *r_n_*} of participant *i* is derived from the trimmed-mean method [26] based on all historical contribution scores of each participant. The trimmed-mean method is a statistical measure of central tendency and involves the calculation of a mean value after discarding given parts of a probability distribution or sample at the high and low end and typically discarding an equal amount of both.


**Algorithm 2:** Accumulated reputation model.
**Input:** Number of participants *N* = {1, 2, …, *n*}, sensing data *S* = {*s*_1_, *s*_2_, …, *s_n_*} of *n* participants**Output:** Reputation score *R* = {*r*_1_, *r*_2_, …, *r_n_*}, sensing result *V***1****for**
*i* = 1 *to n*
**do****2** Computing *M* = {*m*_1_, *m*_2_, …, *m_n_*} using Equations (1) and (2)**3****end****4****for**
*i* = 1 *to n*
**do****5** Computing *C* = {*c*_1_, *c*_2_, …, *c_n_*} using Equations (3) and (4)**6** Computing *R* = {*r*_1_, *r*_2_, …, *r_n_*} using Equation (5)**7****end****8**Computing *V* using Equation (6)


The calculation of *r_i_* is depicted in Equation (5).

(5) $$r_{i}=\frac{c_{i,[n\sigma ]+1}+c_{i,[n\sigma ]+2}+\cdots +c_{i,n-[n\sigma ]}}{k-2[k\sigma ]}$$

where *k* represents the number of observations and *σ* is the coefficient, which is between 0 and 1/2.

The sensing data will be weighted in proportion to the reputation score, *r_i_*. Hence, we can obtain the final sensing result through Equation (6). Generally, procedures of ARM are elaborated in Algorithm 2.

(6) $$V=\overset{n}{\underset{i=1}{\text{∑}}}r_{i}\times s_{i}$$

## 4. Performance Evaluation

In this section, we elaborate the steps taken to evaluate the effectiveness of ARM. We describe the simulation setup in Section 4.1. In Sections 4.2 and 4.3, we present results of our various simulation scenarios, respectively. We also consider the algorithm proposed in [21], which detected inaccurate noise data, and the mechanism proposed in [27], which can be against bad mouthing attack to the reputation system. We take these algorithms for comparison to evaluate the performance of ARM in Section 4.4.

### 4.1. Simulation Setup

This section describes the simulation setup. Considering the participatory sensing application we conduct in our work, participants receive sensing requests from the server and monitor the environment with their devices, then upload sensing data through the Internet; a WiFi connection or a 3G network.

In this work, we simulate a PM2.5 concentration monitoring application using a participatory sensing paradigm. A vector of random values is generated for each participant to represent the PM2.5 concentration value monitored at a specific location in a short time period.

To simulate real scenarios, we classify the participants into three categories: normal participant, inexperienced participant and malicious participant.

Normal participants mostly upload sensing data, which is approximate to the real value in each participatory sensing application. Inexperienced participants are supposed to provide invalid data, due to misuse of devices (kept in a pocket or a bag or the participants are in the buildings) in several applications. In such cases, corrupted PM2.5 concentration data might be recorded by the devices, due to the inadequate propagation of air. More specifically, inexperienced participants are simulated to provide unreliable data in almost half of the sensing applications.

Malicious participants are supposed to intentionally provide corrupted sensing data. We assume these devices are not placed in the right position for the entire duration of sensing, thus contributing to corrupted data. Further, we assume that malicious participants are sophisticated attackers, who have modified the software or sensing results for some reasons, which will introduce negative interference to the final value.

**Figure 3.**
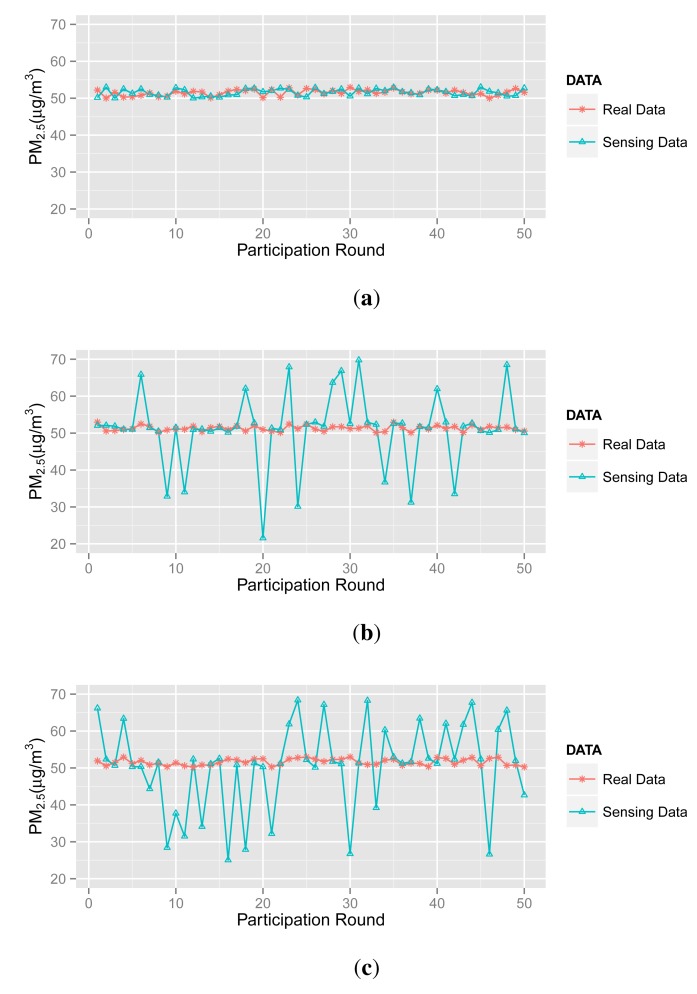
The three kinds of participant. (**a**) Normal Participant; (**b**) Inexperienced Participant; (c) Malicious Participant.

In the simulation, there are 50 participatory sensing rounds. That is to say each participant joins in the application 50 times. The PM2.5 concentration values captured by three types of participants are probably in 50 acts of participation, as shown in Figure 3. Three scenarios (marked as Scenarios A, B, C and D) are adopted in simulations, and forty participants are involved in each scenario. Table 2 illustrates the setups.

In Scenario A, most participants are normal ones, and a small number of abnormal participants upload corrupted sensing data to the server. Note that the abnormal participants are divided into inexperienced ones and malicious ones.

In Scenario B, we define 30 normal participants, seven inexperienced participants and three malicious participants.

In Scenario C, there are only 20 normal participants returning accurate data (actually, this is an extreme circumstance in the real world), 15 inexperienced participants and five malicious participants.

In Scenario D, the numbers of abnormal and normal participants are in equivalent. Additionally, we draw inspiration from the bad mouthing attack [27] and assume that the malicious participants will collude with each other to reduce the reputation of normal participants.

What we consider as common knowledge is that malicious participants are deemed to be a minority in the real world, so only a small amount of malicious participants are defined in each scenario. Even in some extreme environments, like Scenarios C and D, the number of abnormal participant is equal to the number of normal ones.

The setups are used in the following simulations, unless they are specified otherwise.

### 4.2. Sensing Data vs. Contribution Score

#### 4.2.1. Scenario A

**Figure 4.**
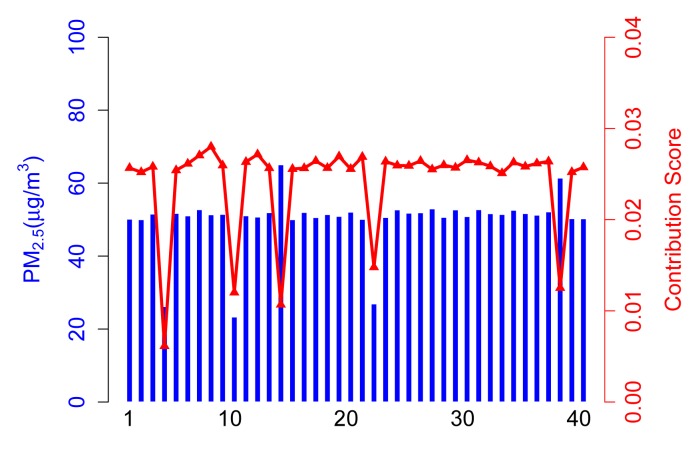
Sensing data *vs.* contribution score in Scenario A.

The contribution score, *C_i_*, in Scenario A is calculated from the *contribution* part of ARM. As shown in Figure 4, 35 normal participants have a relatively higher contribution score than abnormal participants, because they take a majority of the total. It is arduous to get enough of a contribution score for participants who upload corrupted data no matter if the values are higher (see Participant 14) or lower (see Participant 4).

In this case, the contribution score is an exciting way to kick the abnormal participants out and, hence, achieve a more accurate sensing result.

#### 4.2.2. Scenario B

**Figure 5.**
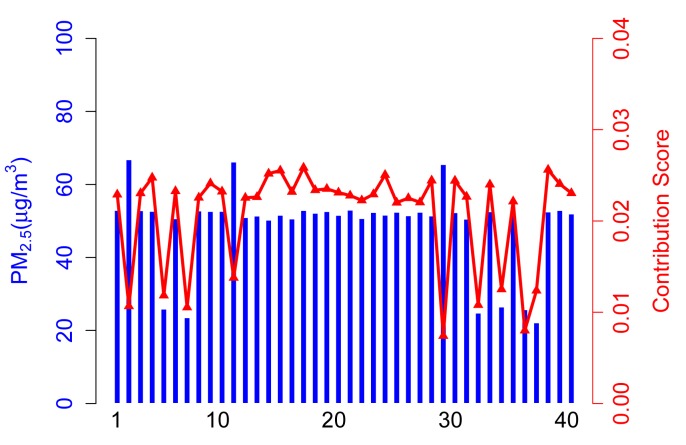
Sensing data *vs.* contribution score in Scenario B.

Figure 5 shows the contribution score and sensing data of each participant in Scenario B.

In this case, there are 30 normal participants. ARM can still identify inexperienced or malicious participants and decrease their contribution score dramatically. Note that there are only nine participants that gain a lower contribution in participation that we selected, because another abnormal participant may provide reliable sensing data in this participation. More specifically, according to the algorithm proposed in preprocessing part, ARM identifies inexperienced or malicious participants, due to normal ones accounting for the majority of total participants in Scenario B. However, abnormal participants take a larger proportion of the total than in Scenario A, and therefore, some abnormal participants get a slightly higher contribution score compared to Scenario A.

#### 4.2.3. Scenario C

**Figure 6.**
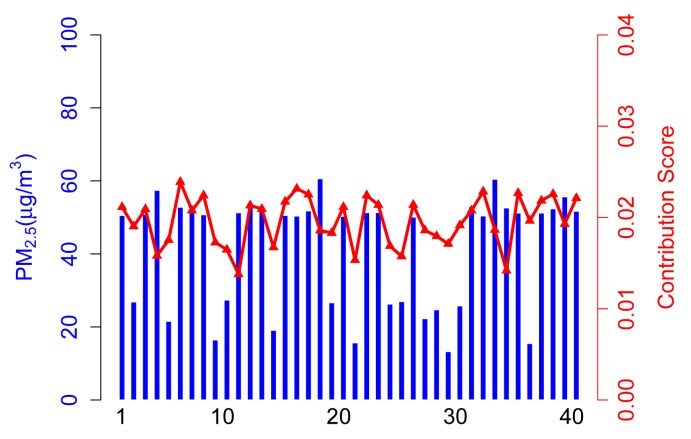
Sensing data *vs.* contribution score in Scenario C.

From Figure 6, abnormal participants take a large proportion of total participants, which will exaggerate the effects of unreliable data in final data calculation. Therefore, normal participants obtain lower contribution scores (see Participants 20, 22, 33, *etc.*). In this extreme case, the sensing result tends to be unreliable.

Note that abnormal participants may provide higher or lower sensing data compared to normal ones, while they will always contribute more to a sensing result if they take the majority. Especially, if a large number of malicious collaborating users take an overwhelming proportion of the total participants, the sensing result may be ridiculous.

#### 4.2.4. Scenario D

**Figure 7.**
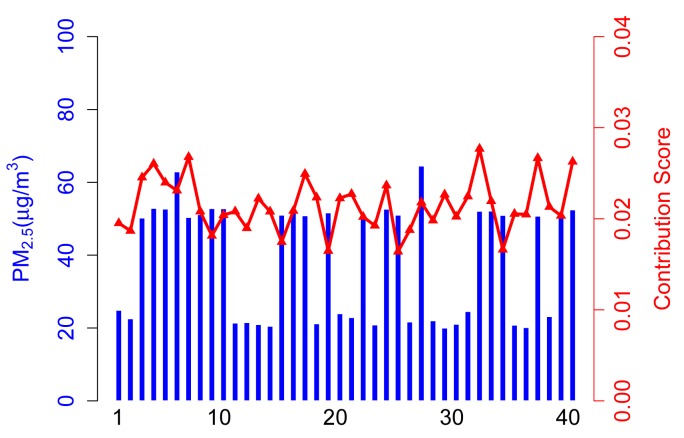
Sensing data *vs.* contribution score in Scenario D.

In this scenario, we assume that abnormal participants, especially malicious ones, collude with each other and always provide inaccurate data to remarkably reduce the quality of sensing results. Figure 7 presents normal participants getting lower contribution scores according to the collusion of malicious participants.

Generally speaking, the contribution part of ARM will efficiently achieve an accurate value if there are only a small proportion of abnormal participants in the application. This makes sense in most participatory sensing scenarios. However, this will lead to unexpected results when abnormal participants get in charge of the system.

### 4.3. Contribution Score vs. Reputation Score

In this section, we compare the contribution score and reputation score of different participants.

More specifically, according to the aforementioned participant types (see Figure 3), we simulate 50 acts of participation under the assumptions defined in Table 3, where normal participants are assumed to contribute accurate data in over 90% of participation, the ratio for inexperienced participants is 50%–60% and malicious participants intend to collapse the application by providing corrupted data in more than 80% of participation. Note that the behaviors of the normal, inexperienced and malicious participants are identical in all four scenarios to demonstrate the tendency of the contribution score and reputation score.

#### 4.3.1. Normal Participant

**Figure 8.**
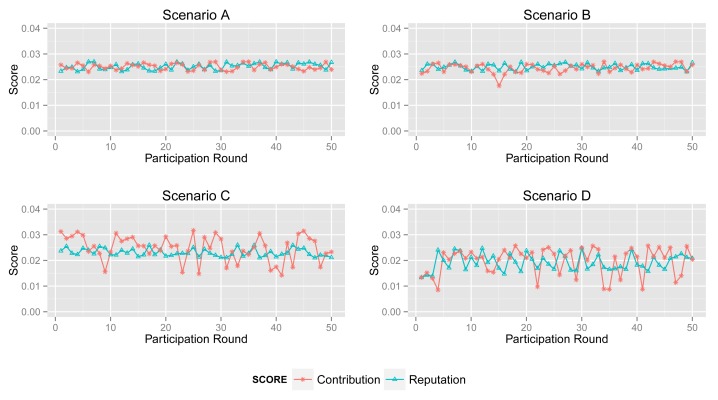
Contribution score *vs.* reputation score of a normal participant in Scenarios A, B, C and D.

Figure 8 shows the contribution score and reputation score of normal participant in Scenarios A, B, C and D.

The normal participant is always one of the majorities who provides accurate sensing data in Scenarios A and B. Therefore, it obtains a high and stable contribution score and reputation score in both scenarios.

In Scenario B, abnormal participants account for a slightly greater proportion than that in Scenario A, although normal ones are still a majority. From the second sub-graph, the normal participant mostly gets a high contribution score. Moreover, its reputation score will not fall down dramatically when its contribution score decreases sharply by providing inaccurate data unintentionally. Obviously, the high reputation score of the normal participant will benefit the entire system. Generally, normal participants usually provide reliable data and also get a high contribution score in most cases.

However, from the third and fourth sub-graphs, the normal one always provides reliable sensing data while it obtains enough of a high contribution score in just a few acts of participation, because abnormal participants account for a large proportion in Scenarios C and D. Particularly, the malicious participants in Scenario D collude with each other to improve their reputation. In this circumstance, the processing and the contribution part of ARM just identify the minority from the total data. Namely, ARM may regard normal participants as malicious ones if normal participants account for a large proportion. More specifically, in some acts of participation, the contribution score of the participant in Scenario B is quite different from that in Scenarios C and D, whereas sensing data in the two scenarios are extremely similar. Nonetheless, the *reputation* part of ARM can track the historical contribution score of each participant to illustrate its behavior in previous participation. The final sub-graph shows that a normal participant's contribution score stays at a relatively high level and increases gradually, though it is lower in some participation.

#### 4.3.2. Inexperienced Participant

**Figure 9.**
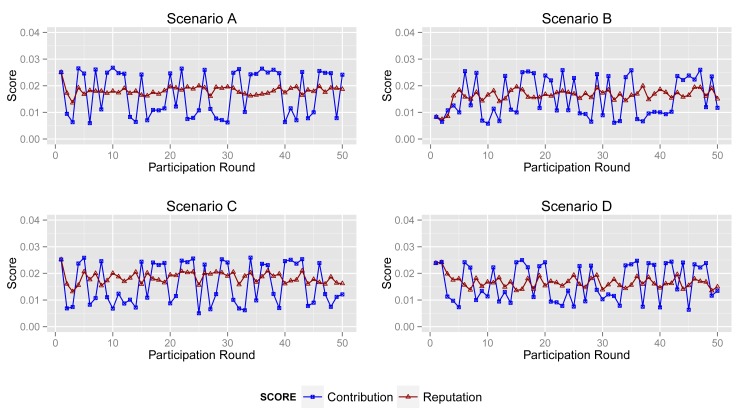
Contribution score *vs.* reputation score of inexperienced participant in Scenarios A, B, C and D.

Figure 9 depicts the contribution score and reputation score of an inexperienced participant in four scenarios. In the participation, they provide corrupted data in about 50% of participation. Hence, ARM decreases its reputation score as a punishment, even though it returns accurate data in several acts of participation.

The second sub-graph illustrates the changing of the contribution and reputation score of an inexperienced participant in Scenario B. It is clear to see that the reputation score reflects the tendency of contribution. Its reputation score falls down when its contribution score decreases acutely and *vice versa*.

A comparison between the reputation score and the contribution score in Scenario C is shown in the third sub-graph. The tendency of its contribution score in the first several acts of participation is also decreasing. Its contribution score is similar with that in Scenario B. However, this is not true after analysis. In Scenario C, there are less normal participants than in Scenarios A and B. Corrupted sensing data may account for the major proportion of all data.

Obviously, the contribution part of ARM accepts the same sensing data, but produces a different contribution score. That is to say that a high contribution score may be led by unreliable data, and a low contribution score exceptionally reflects normal sensing data. However, the *reputation* part of ARM can improve the effect of normal data and decrease the interference from corrupted data.

#### 4.3.3. Malicious Participant

Malicious participants provide unreliable sensing data in most acts of participation, but their contribution scores in four scenarios are remarkably different. Malicious participants mostly return corrupted data in general.

**Figure 10.**
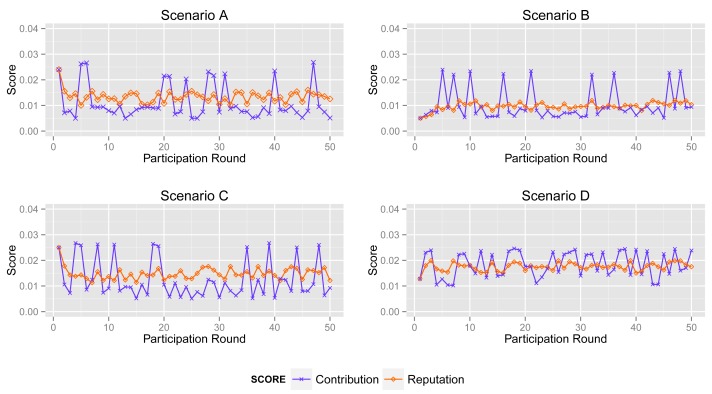
Contribution degree *vs.* reputation score of a malicious participant in Scenarios A, B, C and D.

Shown in the first sub-graph in Figure 10, the malicious participant obtains a low contribution score and reputation score, due to generating corrupted data in most acts of participation. Although they sometimes intentionally provide normal sensing data in order to increase their contribution score and affect the sensing result, ARM will reduce its influence on the result by using its reputation score in calculation.

However, the malicious participant gains a relatively high contribution score in most acts of participation in Scenarios C and D. Apparently, the *processing* and the *contribution* part of ARM cannot play an expected role in circumstances like Scenarios C and D. In Scenario D, the malicious participants, sometimes, have a high probability of obtaining a high contribution, since they are collusive.

Given the reputation score of the malicious participant in four scenarios, the *reputation* part always decreases the reputation score of the malicious participant remarkably. Note that the graph shows that the contribution score and reputation score are quite similar in the first act of participation. Although ARM cannot identify the type of participants when they first join in a sensing application, the model can still identify sensing data and participant's behavior after several acts of participation when it produces a relatively accurate result.

### 4.4. Final Sensing Result

#### 4.4.1. Scenario A

**Figure 11.**
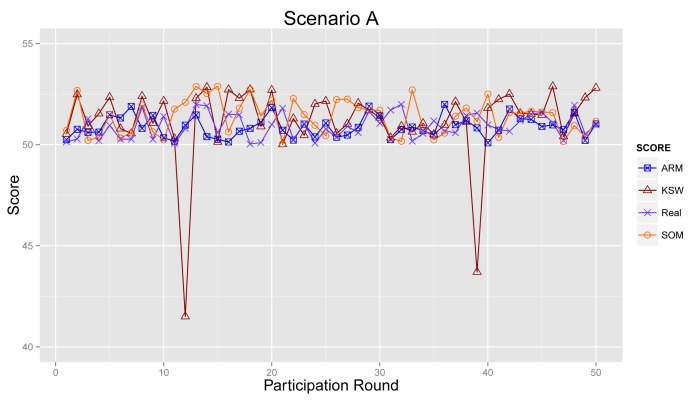
Final sensing result in Scenario A.

Figure 11 plots the sensing results generated by the proposed ARM (marked as ARM), real sensing results in the physical world and values obtained from the algorithm raised in [21] (KSW for short) and [27] (denoted as SOM), respectively.

As shown in Figure 11, sensing results from ARM, KSW and SOM are much more approximate to real values, because most participants are normal ones in Scenario A. There is little bad influence from abnormal participants. The sensing result from KSW becomes ridiculous in participation Round 12 and Round 39, and the results of ARM and SOM can revise this drawback, since these two mechanisms can detect outliers efficiently.

#### 4.4.2. Scenario B

**Figure 12.**
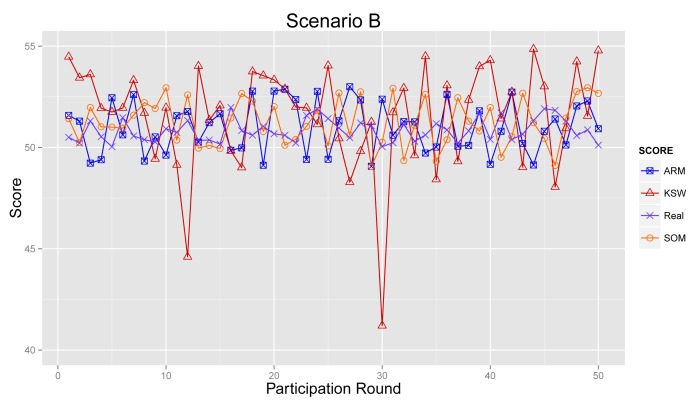
Final sensing result in Scenario B.

Figure 12 illustrates the sensing results in Scenario B. From the graph, we can see that the sensing results from ARM, KSW and SOM deviate slightly further from the real values than in Figure 11, due to a lesser number of normal participants. Further, sensing results from ARM are mildly closer than KSW and SOM.

#### 4.4.3. Scenario C

**Figure 13.**
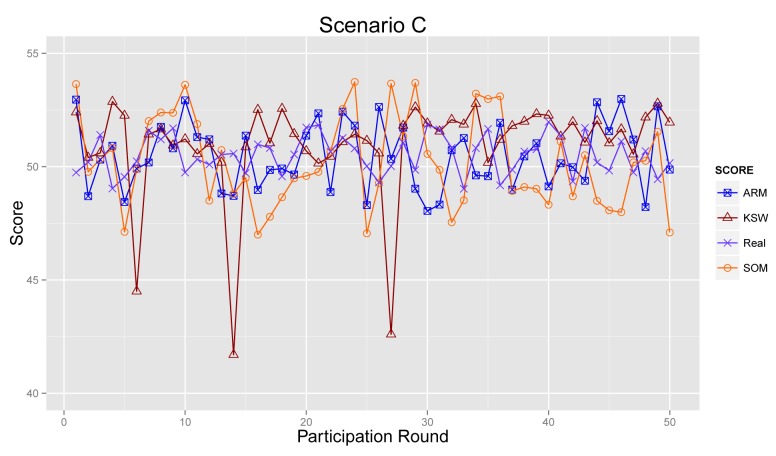
Final sensing result in Scenario C.

Figure 13 provides the sensing results in Scenario C. The ARM model decreases the interference of abnormal participants and increases the effect of accurate data by introducing the reputation score of participants. Obviously, as depicted in Figure 13, the final results of KSW are quite far from real values. By contrast, ARM provides accurate results. SOM also can reduce some of the negative influences of the sensing data from abnormal ones. However, compared with SOM, ARM can obtain more reliable sensing results, through considering historical data and accumulated reputation scores.

#### 4.4.4. Scenario D

**Figure 14.**
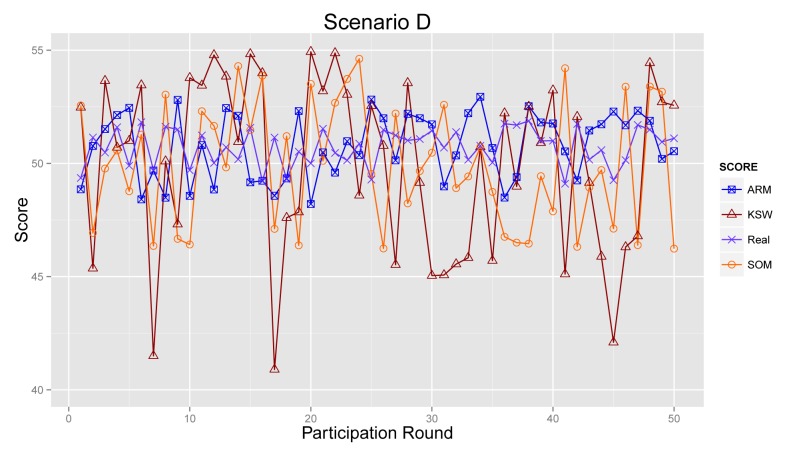
Final sensing result in Scenario D.

As presented in Figure 14, the results from ARM, KSW and SOM fluctuate strongly during 50 acts of participation in Scenario D. Obviously, the result generated by KSW is far away from the real result. For SOM and ARM, they both decrease the effects from corrupt information and improve the accuracy of sensing results. However, based on the accumulated reputation score, ARM can obtain a more reliable sensing result than SOM. To summarize, ARM can produce sensing results that are approximately to real ones, especially in the cases where normal participants did not take the majority of the total.

## 5. Conclusions

We presented ARM, an accumulated reputation model in participatory sensing systems. In light of the sensing data collected by participants, ARM can identify and reduce a bad influence to obtain accurate sensing results. We experimentally evaluated ARM within simulations for PM2.5 concentration monitoring. Furthermore, ARM still produces relatively accurate results in the scenarios with insufficient normal participants. The simulation results show that ARM improves the sensing result quality by impairing the influences of corrupted data. Future study will extend the ARM model to real-world experiments.

## Figures and Tables

**Table 1.** Summary of notations.

**Symbols**	**Definition**
*N*	The number of participants
*S*	The sensing data provided by participants
*M*	The weights of all sensing data
$M_{i}^{f}$	The final weight of sensing data collected by participant *i*
$M_{i}^{\text{norm}}$	The final weight of sensing data collected by participant *i* after normalization
*∊*	A small positive constant to improve the algorithm's numerical properties
*σ*	The coefficient, which is between 0 and $\frac{1}{2}$
*C*	The contribution score of sensing data from participants
*R*	The reputation score of each participant
*V*	The sensing result

**Table 2.** Participant composition in three scenarios.

**Scenario**	**Normal**	**Abnormal**		

		**Inexperienced**	**Malicious**
A	35	3	2
B	30	7	3
C	20	15	5
D	20	0	20

**Table 3.** Sensing data provided by participants.

**Participant**	**Normal Data**	**Corrupted Data**
Normal	90–100%	0–10%
Inexperienced	50–60%	40–50%
Malicious	10–20%	80–90%
